# Improvement of intestinal microbial structure in patients with cerebral infarction through in vitro fermentation of anthocyanins from *Lycium ruthenicum* Murray

**DOI:** 10.1002/fsn3.4263

**Published:** 2024-07-28

**Authors:** Jun Qiu, Bin Ye, Lei Feng

**Affiliations:** ^1^ Stroke Center The Third People's Hospital of Bengbu Bengbu Anhui China; ^2^ Department of Neurosurgery The First People's Hospital of Jining Jining Shandong China; ^3^ Jining Key Laboratory of Stroke and Nerve Repair Jining Shandong China

**Keywords:** anthocyanins, cerebral infarction, fermentation, intestinal microbiota, *Lycium ruthenicum* Murray

## Abstract

Anthocyanins in *Lycium ruthenicum* Murray can be degraded into metabolites by intestinal microorganisms and have a wide range of biological functions. However, there are limited studies on the effect of anthocyanins on the intestinal flora structure in patients with cerebral infarction. To explore the new probiotic effects of ACN, the gut microbiota present in fecal samples obtained from healthy volunteers and patients with acute cerebral infarction underwent in vitro fermentation analysis. The in vitro fermentation product of ACN with *L. ruthenicum* Murray can significantly increase the diversity of the gut flora in patients with cerebral infarction. It can also promote beneficial bacteria (e.g., Bifidobacterium) in the guts of patients with acute cerebral infarction (e.g. Bifidobacterium, Allisonella, and Prevotell), reduce the growth of potentially harmful bacteria (Dialister, Megamonas, and Clostridium), and increase the levels of SCFAs. This investigation demonstrated the capability of ACN in vitro fermentation to improve the gut microbiota structure in patients with cerebral infarction. This, in turn, furnishes new theoretical underpinnings for its potential development as a functional food component.

## Graphical abstract

The in vitro fermentation product of *Lycium ruthenicum* Murray anthocyanins significantly increased the diversity of the intestinal microbiota in patients with cerebral infarction. ACN promoted the proliferation of beneficial bacteria and decreased the growth of potentially harmful bacteria in the gut of patients with acute cerebral infarction. ACN increased the content of SCFAs.

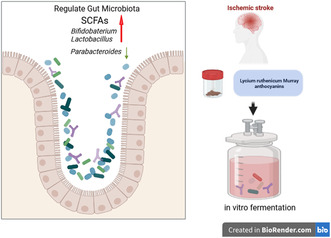

## 1 INTRODUCTION

It is estimated that there are approximately 10^14^ bacterial cells in the gut microbiome, which is around ten times the total number of human somatic cells. Consequently, the gut microbiota constitutes an intricate microbial ecosystem pivotal to human well‐being and disease (Chen, Xu, et al., 2019; Clemente et al., 2012). Cerebral infarction is a serious neurological disease whose incidence continues to increase worldwide (Xu et al., 2021). The onset and progression of cerebral infarction involve several complex physiological and pathological processes, among which the gut flora imbalance has recently been gradually recognized and has attracted much attention from researchers (Cryan et al., 2020). Recent research suggests a correlation between gut microbiota and the incidence and recuperation of cerebral infarction (Peng et al., 2023). There is a complex interaction between the nervous system and the gut, known as the gut‐brain axis (Tremlett et al., 2017). Gut flora can influence nervous system function through multiple pathways (Chen, Xu, et al., 2019). The gut microbiota can affect neuronal activity and excitability by generating metabolites, including short‐chain fatty acids (SCFAs), amino acids, and neurotransmitters. Secondly, it can impact neuroinflammation by regulating immune system function (Zhang et al., 2021). Gut flora can influence immune cells' inflammatory response and neuroinflammation development by inducing immune cells' differentiation and functional regulation (Rothhammer et al., 2016; Yin et al., 2015). Furthermore, diet is widely acknowledged as a crucial influencer of gut microbe establishment and composition across the life cycle. Therefore, shaping and maintaining healthy gut microbes is important (Puupponen‐Pimiä et al., 2005). For example, dietary polyphenols can promote beneficial bacteria (e.g. Bifidobacterium and Lactobacillus) while inhibiting pathogenic bacteria (e.g. Helicobacter, Staphylococcus, Bacillus cereus, and Salmonella) (Nohynek et al., 2006). This leads to a change in the composition of gut microbes within the human body. Dietary polyphenols, particularly anthocyanins, are therefore widely considered to regulate gut microbes (Jamar et al., 2017; Liang et al., 2023).

*Lycium ruthenicum* Murray is a plant that grows at high altitudes and is widely used in the field of health care and the treatment of many diseases (Wang, Fang, et al., 2018). It has been reported that Lycium ruthenium Murray is rich in anthocyanosides, which have a wide range of biological functions, including anti‐radiation, anti‐oxidant, anti‐inflammatory, anti‐tumor, diabetes, and cardiovascular risk for a variety of chronic diseases (Shin et al., 2006; Wang, Li, et al., 2018; Wu et al., 2016). However, limited research exists on *L. ruthenicum* Murray's impact on stroke patients' gut microbiota configuration. Studies have found changes in the composition and species of intestinal flora after cerebral infarction (Stanley et al., 2018), disruption of intestinal epithelial integrity (Stanley et al., 2016), and abnormalities in intestinal immune function (Singh et al., 2016). However, most of this evidence is derived from animal research models and lacks clinical trial data. This paper aims to examine the modulatory impact of in vitro fermentation of anthocyanins from *L. ruthenicum* Murray on the composition of intestinal microflora in patients with cerebral infarction. We will select fecal samples from patients with clinically confirmed cerebral infarction as a basis for the initial assessment of gut flora while using in vitro fermentation techniques to process *L. ruthenicum* Murray anthocyanosides to obtain products with better bioavailability and activity. By analyzing the fermentation products and evaluating their effects on gut flora structure, we will determine whether, in vitro fermentation of *L. ruthenicum* Murray, anthocyanosides can improve gut flora structure in patients with cerebral infarction, thus providing new theoretical support for its development as a functional food ingredient.

## 2 MATERIALS AND METHODS

### 2.1 Study population

Patients with acute cerebral infarction were admitted to the First People's Hospital of Jining City for treatment in 2023, and the control population of the physical examination center in the same period.

### 2.2 Inclusion and exclusion criteria

Inclusion criteria for the case group are as follows: (1) subjects have signed the informed consent; (2) meet the diagnostic criteria for acute cerebral infarction in the Chinese Guidelines for the Diagnosis and Treatment of Acute Ischemic Cerebral Infarction (2018 edition) (Wang, Fang, et al., 2018); (3) age ≥18 years; and (4) lipid and blood glucose in the normal range. Exclusion criteria for the case group are as follows: (1) combination of other diseases: neurological diseases (previous cerebral infarction, cerebral hemorrhage, multiple sclerosis, etc.); all kinds of chronic digestive diseases, with acute digestive diseases within three months; cardiovascular diseases (myocardial infarction, heart failure, and atrial fibrillation); other diseases (mental diseases, food allergies, and tumors); (2) history of blood transfusion, history of surgery for digestive diseases, and history of trauma; (3) use of the following drugs within three months: Antibiotics, Laxatives, Clonazepam, Mesalazine, TNF‐α Inhibitors, Immunosuppressants, Antidepressants, PPIs, Opioids, Traditional Chinese Medicines, Chinese Patent Medicines, etc.; (4) use of probiotic preparations within one month; (5) use of antiplatelet and statin drugs before current disease onset; (6) patients who underwent intravenous thrombolysis and endovascular interventional therapy; and (7) pregnant or lactating women. Inclusion criteria for the control group are as follows: (1) subjects had signed informed consent; (2) age ≥18 years; and (3) lipid and blood glucose in normal range. We incorporated a total of six people. They were divided into 3 people in the healthy control group (original stool group, OR group) and 3 people in the group of patients with acute cerebral infarction (ORI group).

### 2.3 Preparation of anthocyanins

The preparation of anthocyanins was based on the reported method with some modifications (Ravanfar et al., 2016). In brief, the dried fruit of the black wolfberry is taken, and an ethanol aqueous solution (80%) contains 0.1% formic acid. Mix the two in a ratio of 1:40 and perform the extraction in a water bath at 50°C for 3 h each time. Filter the extract with gauze and concentrate it through a vacuum rotary evaporator. Next, the concentrated liquid is loaded onto AB‐8 macroporous adsorption resin for further processing. First, highly polar components were eliminated and washed with two‐column volumes of deionized water. Next, an 80% ethanol solution (with 0.1% formic acid) was used at a 2.0 mL/min flow rate to elute the target component (anthocyanins). Finally, the eluent was concentrated, freeze‐dried, and yielded the anthocyanin powder utilized for the experiment (Peng et al., 2019).

### 2.4 In vitro fermentation

A previously reported method, with some modifications, was followed to simulate colonic fermentation (Xie et al., 2017). For the experiment, fresh feces were collected from three healthy volunteers and three patients with acute stroke; the healthy volunteers had not taken antibiotics for at least three months. A sterile modified saline solution containing 8.5 g/L NaCl and 0.5 g/L cysteine hydrochloride was prepared. The solution was diluted by a factor of 10 and mixed well, placed at 4°C, and then centrifuged for 5 min (500 rpm). The supernatant obtained was used as a fecal slurry for subsequent fermentation, and the microbial community composition of the fecal slurry was recorded as the OR group. We prepared basic nutrient growth medium (peptone 2.0 g/L, yeast extract 2.0 g/L, NaCl 0.1 g/ L, K_2_HPO_4_ 0.04 g/L, MgSO_4_ 0.01 g/L, CaCl_2_ 0.01 g/L, NaHCO_3_ 2.0 g/L, hemim 0.02 g/L, cysteamine hydrochloride 0.5 g/L, bile salts 0.5 g/L, blades of azure 1.0 mg/L, tween 80 2.0 mL/L, and vitamin K1 10 mL/L, bladed azurite 1.0 mg/ L, tween 80 2.0 mL/L, and vitamin K1 10 μL/L). The basal nutrient growth medium was autoclaved, and the anthocyanins (1.0 g/L) were dissolved. Further, 1.0 mL of varied slurry suspensions were introduced into the vegetative growth medium containing anthocyanins. Afterwards, the triangular vials were positioned in the Anaero Pack system for in vitro fermentation at a temperature of 37°C for a culture period of 24 h. To maintain the oxygen pressure below 0.1% in the sealed chamber, including an oxygen indicator is imperative. During the fermentation period, the chamber was gently shaken every 6 h. Finally, the fermented samples were removed for further experimental studies.

### 2.5 Quantification of anthocyanin during in vitro fermentation

An Agilent 1200 Infinity HPLC equipped with a binary pump and a diode array detector was used for the quantitative determination of *L. ruthenicum* Murray ACN.

### 2.6 Determination of short‐chain fatty acids and lactic acid content

The content of SCFAs in the fermentation broth of different groups was determined via a gas chromatography‐flame ionization detector (GC‐FID), according to the method outlined in the literature (Patel et al., 2012). The SCFAs detected were acetic acid, propionic acid, n‐butyric acid, isobutyric acid, n‐valeric acid, and isovaleric acid. Before determination and analysis, acidifying the samples with an HCl solution was necessary. A lactic acid assay kit was employed, and the manufacturer's instructions were followed to determine the lactic acid concentration.

### 2.7 Analysis of gut microorganisms

The three groups of fermentation broths included the original feces group (OR group), the acute cerebral infarction patient group (ORI group), and the *Lycium barbarum* anthocyanin addition group (LCI group). After 24 h of fermentation, DNA was extracted from the bacteria in the fermentation medium. The extraction process was done using the TIANamp Stool DNA Kit according to the instructions. The DNA samples were sent to Shanghai Tianhao Biotechnology Co., Ltd. for 16S rDNA gene sequencing under −20°C storage and dry ice conditions. The DADA2 algorithm was used for pre‐processing and quality control of all samples. This included the removal of repetitive sequences, denoising, sequence splicing, generation of amplicon sequence variants (ASVs) tables, removal of chimeras, and finally species annotation. Mothur software was used to compare the RDP database and annotate the ASV tables based on the species information where the ASVs were most similar and had a confidence level of 80% or more. The species composition and abundance were counted for each sample at each taxonomic level. Dilution curves and α‐diversity were calculated using Mothur software analysis (Schmidt et al., 2010). For β‐diversity analysis based on the level of ASVs, we used the R language Vegan package (V3.4.0) for statistical analysis. LEfSe was then used to analyze the differential ASVs between different groups of samples.

### 2.8 pH measurement

At the end of anaerobic fermentation, the pH of the culture solution was measured for all culture systems.

### 2.9 Statistical analysis

A one‐way analysis of variance (ANOVA) utilized IBM SPSS 22.0 software. Experiments were repeated thrice, and results were presented as mean ± SD. A *t*‐test was used for normal continuous variables. Comparisons between two groups were made using the Wilcoxon rank‐sum test and between multiple groups using the Kruskal‐Wallis rank‐sum test. Statistical significance was considered at *p* < .05.

## 3 RESULTS

### 3.1 Overall differences in gut microbiology due to in vitro fermentation

**FIGURE 1:**
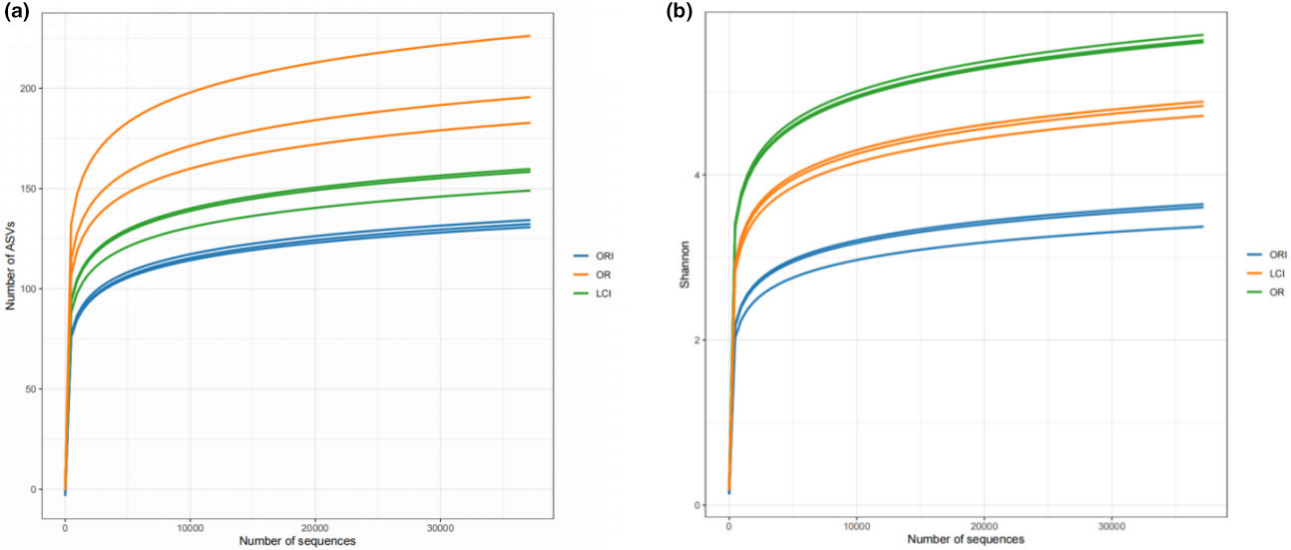
Sample rarefaction curves (a) and Shannon curves (b) for each treatment group. The ORI group has the lowest diversity. The ORI group has the lowest diversity. The OR group has the highest diversity, and the LCI group is in between.

Gut flora, which plays an important role in human well‐being and disease, can be modulated by a daily diet (Zou et al., 2011). Therefore, the present study was proposed to investigate the effect of *L. ruthenicum* Murray anthocyanosides on adult gut flora through in vitro fermentation. This study used high‐throughput 16S rRNA sequencing to analyze nine samples (3 groups, three replicates in each group). The dilution curves of the samples and the curve of the Shannon index are shown in Figure 1, which shows that the number of ASVs continued to increase as the number of reads sampled increased and that the Shannon index tended to be stable, indicating that almost all species were similar to the same ASV. The figure shows that the number of ASVs continuously increased as the number of reads increased and that the Shannon index stabilized, indicating that almost all lineages were covered and that it can truly reflect the species information of most of the species in the sample. In general, the Shannon index is related to diversity. As can be seen in the figure, the ORI group has the lowest diversity. The OR group has the highest diversity, and the LCI group is in between. Studies have shown that the variety of gut microbiota is inversely related to illness vulnerability (Chassaing & Darfeuille‐Michaud, 2011; Mohamadzadeh et al., 2011). Therefore, *L. ruthenicum* Murray anthocyanosides have a better effect on maintaining the diversity of the gut flora and gut health.

**FIGURE 2:**
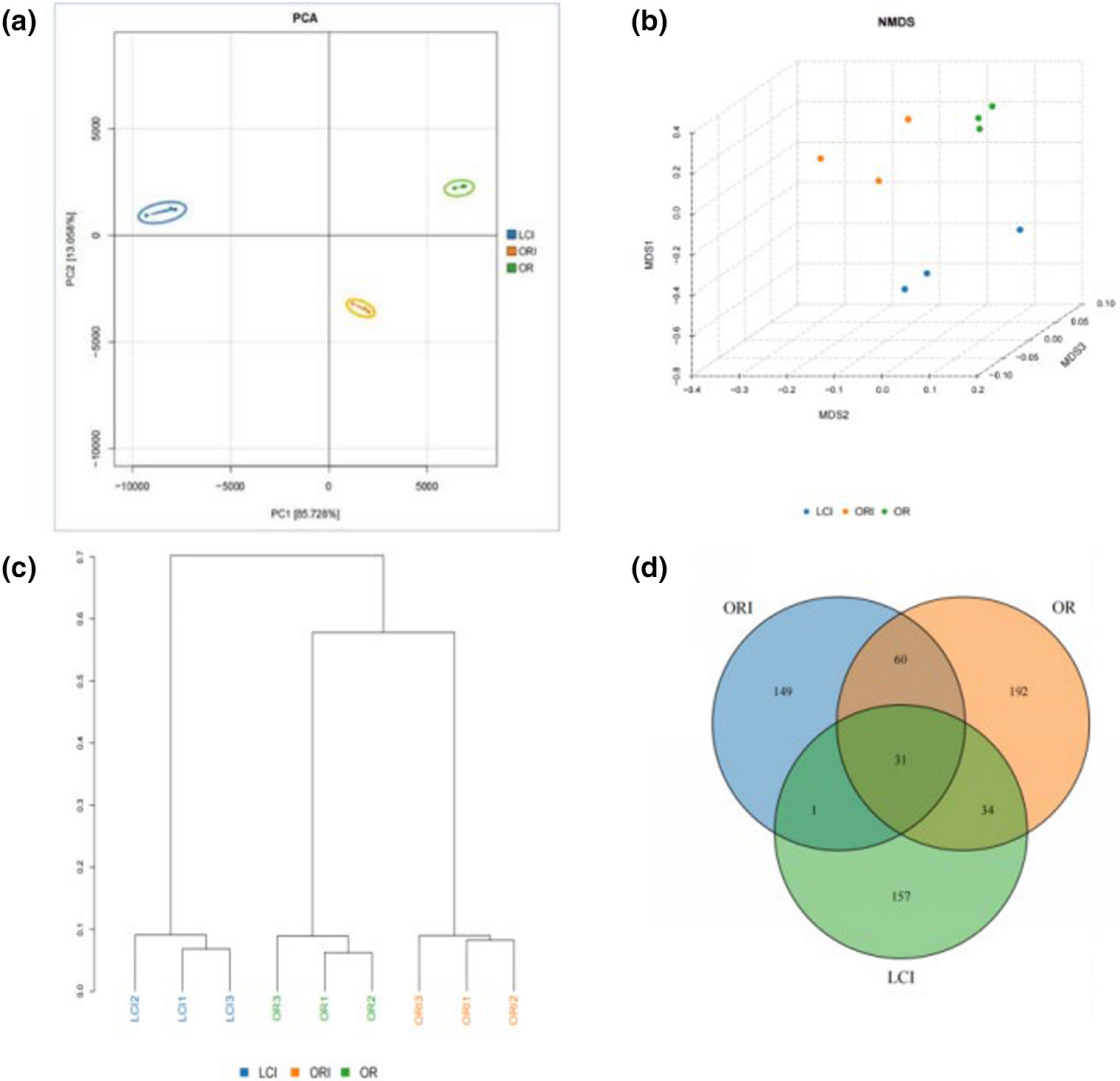
Comparison of overall bacterial composition among groups: PCA (a), NMDS (b), cluster analysis (c), and Venn diagrams (d).

Principal component analysis (PCA) and cluster analysis were employed to compare dissimilarities in microbial community composition between diverse sample groups at the ASV level. As shown in Figure 2a, the PCA plot with the PC1 and PC2 axes explained 98.8% of the total between‐group differences in the in vitro fermentation microflora, suggesting a clear separation and significant differences between groups. Furthermore, the NMDS analysis (Figure 2b) showed similar results. Meanwhile, the cluster analysis results in Figure 2c also showed that the effects on the gut flora varied significantly between groups and were comparable between groups within each group. The Venn diagram (Figure 2d) results showed that the number of ASVs in the OR, ORI, and LCI groups was 192, 149, and 157, respectively, and that 31 common ASVs existed in the three groups simultaneously.

### 3.2 Effect of ACN on gut microbiota

**FIGURE 3:**
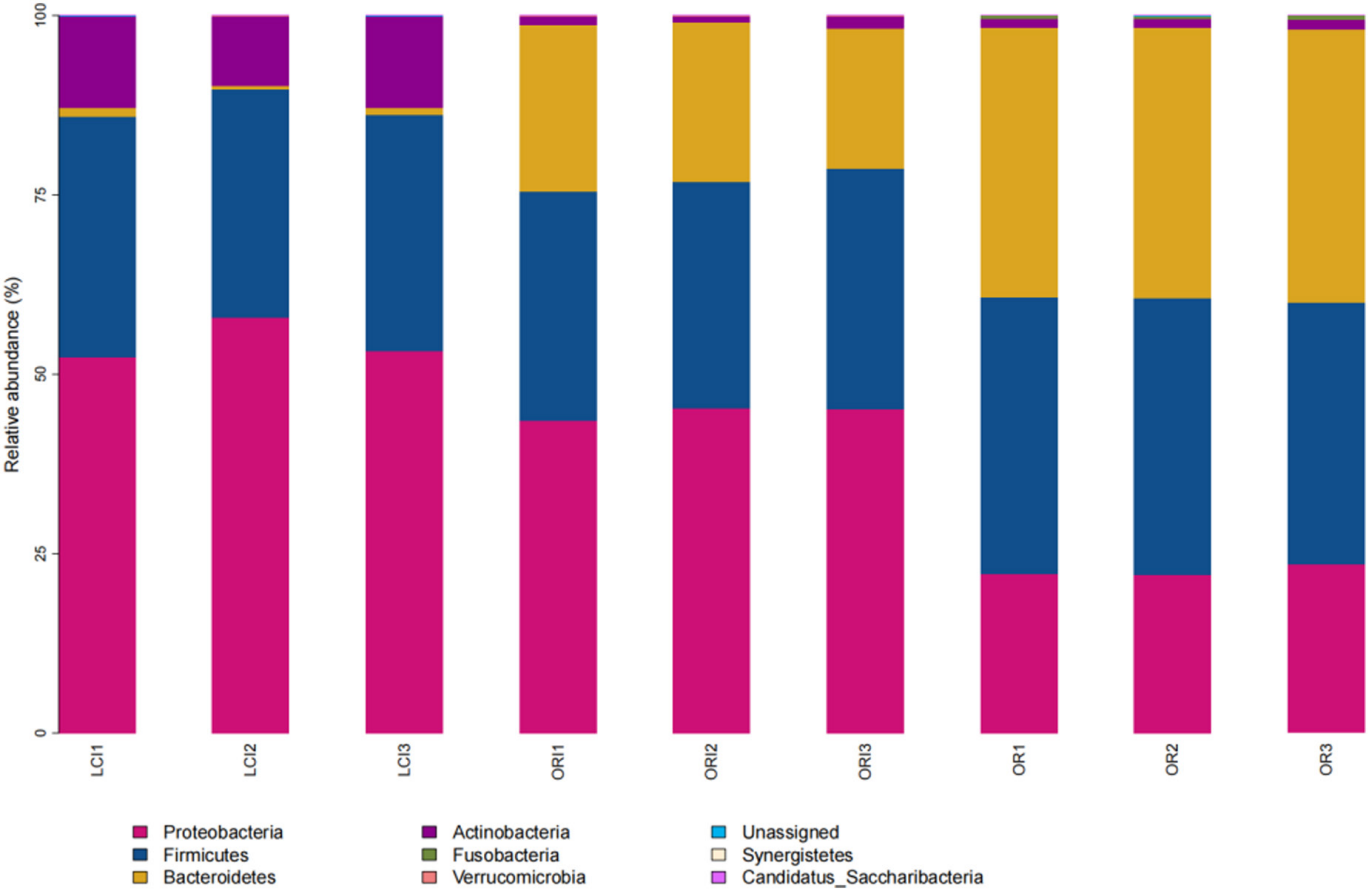
Microbial compositions of the OR group, ORI group, and LCI group at the phylum level. The gut flora composition of samples from each group at the phylum level, with microorganisms from Proteobacteria, Firmicutes, Bacteroidetes, and Actinobacteria phyla forming the majority.

Figure 3 displays the gut flora composition of samples from each group at the phylum level, with microorganisms from Proteobacteria, Firmicutes, Bacteroidetes, and Actinobacteria phyla forming the majority. Differences in phylum abundance among groups were found, with the Actinobacteria phylum being significantly more abundant in the LCI group than the OR group (*p* < .05). Furthermore, there was a significant decrease in the Bacteroidetes phylum within the LCI group compared to the OR group.

**FIGURE 4:**
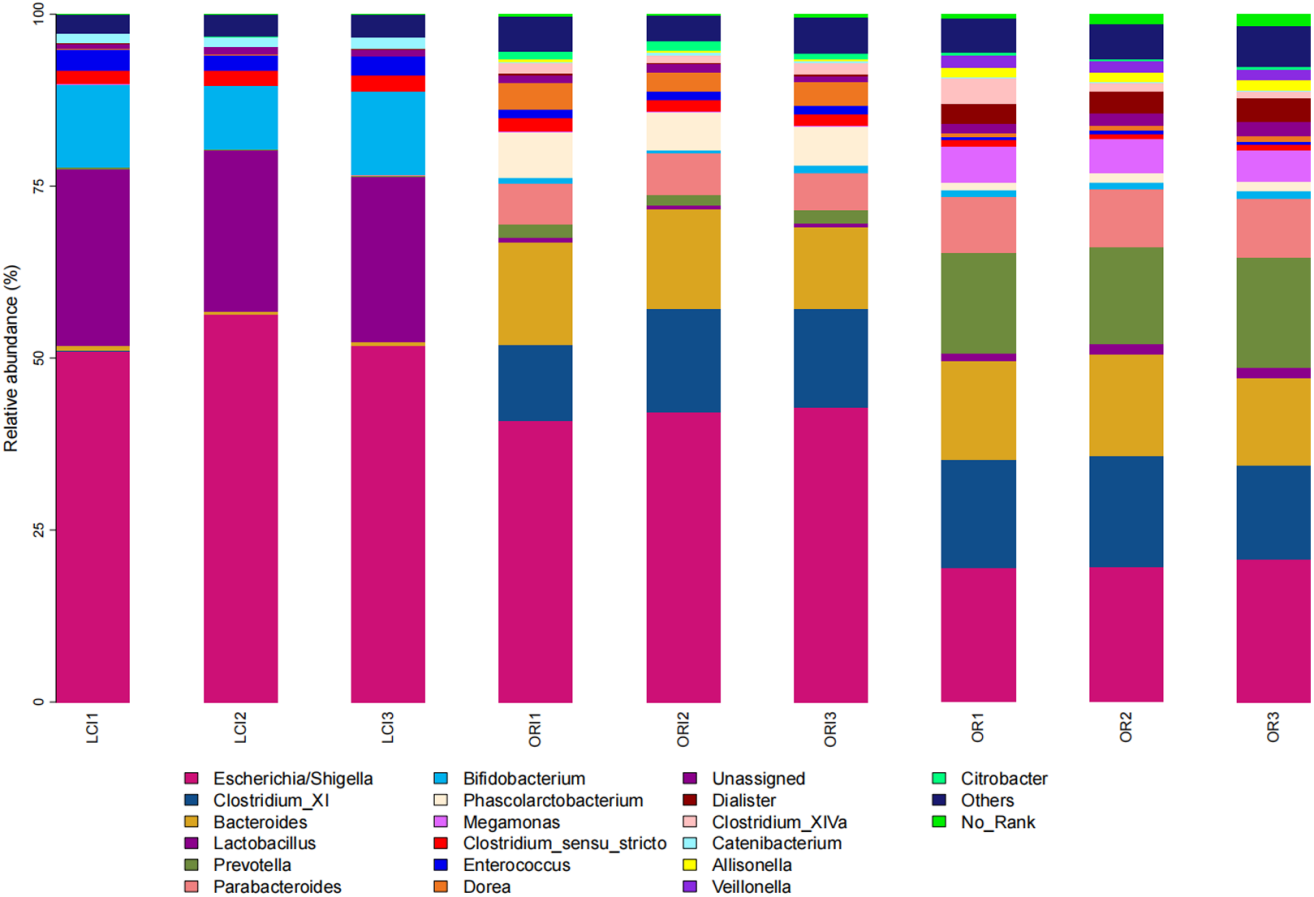
Column diagram of microbial composition at the genus level. The genera Bacteroides, Bifidobacterium, Clostridium XI, Escherichia/Shigella, Megamonas, Lactobacillus, Prevotella, Alistipes, Parabacteroides, Dialister, and Clostridium XIVa were the major microbial taxa in the in vitro anaerobic fermentation system.

To clarify the differences between the groups of samples, we statistically analyzed the genus in which each sample was found. After retaining the genera in which the average abundance of ASV was greater than 0.5% in each group, the genus distribution of the enteric flora in each group of samples is shown in Figure 4; the genera Bacteroides, Bifidobacterium, Clostridium_XI, Escherichia/Shigella, Megamonas, Lactobacillus, Prevotella, Alistipes, Parabacteroides, Dialister, and Clostridium XIVa were the major microbial taxa in the in vitro anaerobic fermentation system. Regarding variations in the relative abundance of probiotics, introducing *L. ruthenicum* anthocyanins (LCI group) has resulted in a noteworthy increase in Bifidobacterium and Lactobacillus. Moreover, it has shown a decrease in the relative abundance of Dialister, Megamonas, and Parabacteroides (*p* < .05).

**FIGURE 5:**
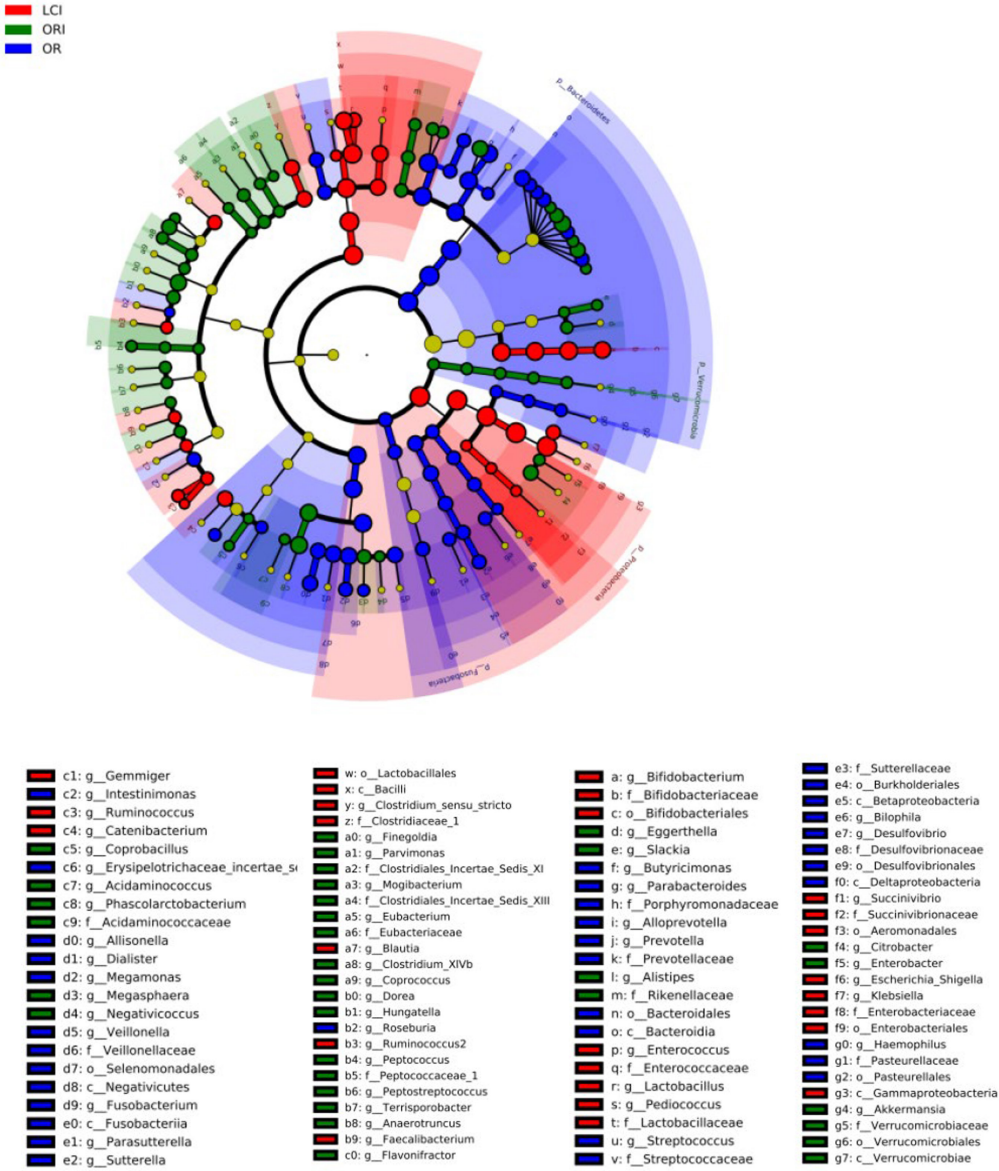
Pilot results of LeFSe analysis at the genus level.

The treatment groups' differences were analyzed based on the data depicted in Figure 5. It reveals that the abundance of bacteria among the groups was significantly higher in specific treatment groups, as highlighted by the differently colored nodes in the branches. According to the preliminary results of the LeFSe analysis, Bifidobacterium, Ruminococcus, and Lactobacillus were the dominant genera in the ACN group.

### 3.3 Effect of ACN on the pH of an in vitro anaerobic fermentation system

Most of the indigestible substances are degraded by microorganisms in the gut, from which the carbon source can be broken down and utilized by the gut flora to produce organic acids. Carbon sources can be broken down and used by intestinal microorganisms to produce organic acids. At the same time, proteins may be deaminated or decarboxylated by these microorganisms to generate compounds, including short‐chain fatty acids, branched‐chain fatty acids, ammonia, and organic amines (Picton et al., 2007; Roediger et al., 1997), and these complex microbial metabolisms are closely related to gut pH. Therefore, to investigate the effect of *L. ruthenicum* Murray anthocyanosides on intestinal microbial metabolism, we determined the pH values of the sample groups with different treatments. As shown in Table 1, after 24 h of fermentation under anaerobic conditions, the LCI group showed the most significant decrease, which may be related to the acid‐producing capacity of Lactobacillus during the fermentation process (Kimura, 2014).

**TABLE 1:** pH of fermentation samples in vitro.

Samples	pH	
	Before fermentation	After fermentation
OR (*n* = 3)	7.46 ± 0.04^Aa^ *	
ORI (*n* = 3)	7.37 ± 0.03^A^	4.11 ± 0.04^b^
LCI (*n* = 3)	7.52 ± 0.02^A^	3.88 ± 0.03^c^

* Different letters indicate significant differences between sample groups (*p* < .05).

### 3.4 Effect of ACN on the production of SCFAs

SCFAs are volatile fatty acids produced by the gut flora and are fermentation products in the large intestine from undigested material from the small intestine (Morais et al., 2016). They can be used as energy stores and reduce intestinal osmotic pressure after rapid absorption, which is important in maintaining normal colonic function and colonic epithelial cell morphology (Hamer et al., 2008). Common SCFAs include mainly acetic acid, lactic acid, propionic acid, n‐butyric acid, isobutyric acid, n‐valeric acid, and isovaleric acid. Therefore, we analyzed the effect of *L. ruthenicum* Murray ACN on the gut flora from a metabolite point of view, using GC to determine the changes in the content of SCFAs during the fermentation process. As shown in Table 2, lactic acid, acetic acid, and propionic acid were the main fermentation products in each treatment. We observed that the LCI group recorded the highest quantity of lactic acid (7.55 ± 0.16 mM). This could be potentially linked to the finding that *L. ruthenicum* anthocyanins considerably augmented the relative abundance of Lactobacillus. Overall, the total acid content correlated with the pH of the culture system, and although acetic acid and lactic acid had higher pKa, the higher the total acid content, the lower the overall pH.

**TABLE 2:** The concentration of SCFAs at different groups during the fermentation.

Groups	SCFAs (mmol/L)							
	Acetic acid	Lactic acid	Propionic acid	i‐Butyric acid	Butyric acid	i‐Valeric acid	Valeric acid	Total
OR (*n* = 3)	0.33 ± 0.07^d^ *	0.25 ± 0.03^d^	0.00 ± 0.00^c^	0.00 ± 0.00^c^	0.10 ± 0.03^c^	0.00 ± 0.00^c^	0.00 ± 0.00^b^	0.68 ± 0.13^d^
ORI (*n* = 3)	7.26 ± 0.18^c^	3.23 ± 0.14^b^	7.33 ± 0.28^a^	0.00 ± 0.00^c^	0.36 ± 0.07^b^	0.10 ± 0.03^b^	0.26 ± 0.10^a^	18.54 ± 0.80^b^
LCI (*n* = 3)	9.56 ± 0.07^b^	7.55 ± 0.16^a^	2.07 ± 0.12^b^	0.33 ± 0.07^a^	0.50 ± 0.15^b^	0.26 ± 0.10^a^	0.00 ± 0.00^b^	20.27 ± 0.67^a^

* Different letters in the same column indicate significant differences between sample groups (*p* < .05).

### 3.5 Impacts of in vitro fermentation on the ACN profile

**FIGURE 6:**
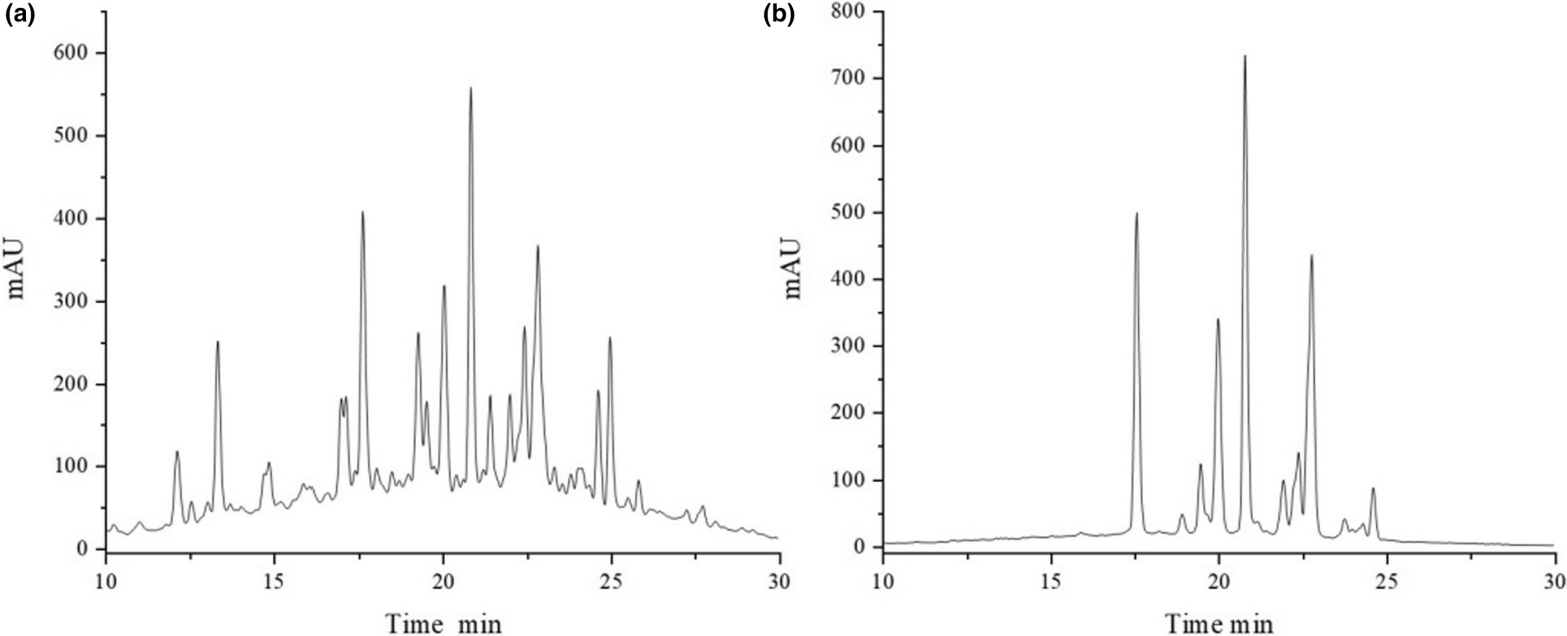
HPLC chromatograms of anthocyanosides of *Lycium ruthenicum* Murray before (a) and after (b) fermentation. The content of anthocyanin monomers decreased with fermentation time due to the gut microbiota.

Given the fact that anthocyanins from *L. ruthenicum* Murray are not readily absorbed during digestion, their catabolism after reaching the colon is largely dependent on the intestinal microbiota. In order to clarify the fermentation characteristics of *L. ruthenicum* Murray anthocyanins, the content of anthocyanin monomers before and after fermentation was determined. Due to the insufficient content of anthocyanins, it was difficult to quantify certain monomers, despite the presence of peaks in the HPLC chromatograms (Figure 6). However, it can be seen that the content of anthocyanin monomers decreased with fermentation time due to the action of the intestinal microbiota.

## 4 DISCUSSION

The results of this study imply that the sequencing depth is adequate and the sequencing outcomes are trustworthy. This inference is deduced from the sample dilution profile, as indicated by the Shannon curve results. Only a small portion of anthocyanin is absorbed in the oral cavity, stomach, and small intestine in the human body; most of it is unabsorbed and reaches the colon, where it interacts with the intestinal microbiota, thus possibly contributing to the low bioavailability of anthocyanin (Zhang et al., 2017). This may be why ACN shows stability in the upper gastrointestinal tract. The content of anthocyanin monomers decreased with fermentation time due to the gut microbiota (Figure 6). In this experiment, we investigated the effect of ACN from *L. ruthenicum* Murray on intestinal microorganisms in patients with cerebral infarction. The principal component analysis and cluster analysis results have demonstrated that ACN extracted from *L. ruthenicum* Murray can potentially regulate the gut microbiota of patients with cerebral infarction. The results also suggest ACN may be a prebiotic‐like positive modulator of gut microbes. The promotional effect of anthocyanin on Actinobacteria has also been reported in the literature, mainly promoting the proliferation of Bifidobacterium in Actinobacteria (Guglielmetti et al., 2013). This further confirms the probiotic effect of ACN. ACN significantly increased the relative abundance of Bifidobacterium and Lactobacillus compared to the OR group (Figure 3). Although the proportion of Lactobacillus and Bifidobacterium in the human body is small, they contribute significantly to gut health (Chambers et al., 2018). An ACN‐induced increase in Bifidobacterium in *L. ruthenicum* Murray has been reported in the literature (Hidalgo et al., 2012) (Morais et al., 2016). Bifidobacterium has been shown to improve intestinal barrier function, stimulate the immune system, and be involved in calcium absorption, vitamin activation, and regulation of lipid metabolism, among other processes (Kovatcheva‐Datchary et al., 2015). Bifidobacterium is a widely recognized beneficial bacterium and the most important bacteria associated with human gut well‐being (Liang et al., 2023). Lactobacillus plays an important neuroinflammatory mitigating role in ACN, and in vitro experiments have confirmed the proliferative effects of ACN on five species of Lactobacillus (Peng et al., 2023). In addition, Lactobacillus and Bifidobacterium may confer antimicrobial effects on pathogens by competing for substrates and adhesion sites and lowering colonic luminal pH (Drago, 2019). ACN‐induced changes in the relative abundance of gut microorganisms may be caused by anthocyanoside metabolites produced by degradation by gut microorganisms. Bacteria associated with anthocyanin metabolism include Bacteroides, Clostridium, Eubacterium, Ruminococcus, and Eggertheilla (Peng et al., 2020). According to the preliminary results of the LeFSe analysis, Bifidobacterium, Ruminococcus, and Lactobacillus were the dominant genera in the ACN group (Figure 5). Thus, anthocyanin may improve the gut microbiota structure by modulating beneficial flora and promoting the proliferation of flora associated with anthocyanin metabolism. Short‐chain fatty acids, including lactic, acetic, propionic, and butyric acids, are the final results of dietary fiber production via anaerobic fermentation by microorganisms in the gut. These SCFAs are thought to promote fiber intake and contribute to gut health (Peng et al., 2019). Acetic, propionic, and butyric acids were the primary fermentation products. Acetic acid is the final product of Bifidobacterium fermentation, and a rise in its levels could indicate a proportional increase in Bifidobacteria abundance (Sanz et al., 2005). Butyric acid serves as a significant energy source for the colon while also contributing to the health of the colonic mucosa. Additionally, it importantly regulates intracellular gene expression (Lee et al., 2015; Vital et al., 2014). Lactic acid is also a significant acid. Lactic acid is a key intermediate for other short‐chain fatty acids, including acetic acid, propionic acid, and butyric acid. Notably, increases in the latter three are typically correlated with reductions in lactic acid levels (Peng et al., 2019). Overall, ACN from *L. ruthenicum* Murray has similar effects to prebiotics and promotes the production of SCFAs by modulating gut microbes (Liang et al., 2023). Crucially, research across multiple models has discovered that ACN and its colonic metabolites from *L. ruthenicum* Murray can regulate the transformed gut microbiota. This is primarily achieved by stimulating advantageous bacterial growth and inhibiting harmful bacterial expansion (Faria et al., 2014). The study found an increase in the relative abundance of Bifidobacterium, a bacterium associated with acetic acid production. In contrast, the abundance of Dialister and Megamonas decreased. Megamonas is associated with the production of SCFAs, and Dialister mainly produces propionic acid, which may positively correlate with lactate content (Lee et al., 2015) (Vital et al., 2014). Hence, maintaining normal gut function necessitates the maintenance of a gut microbial composition that provides both vitamins and short‐chain fatty acids (SCFAs), as well as immune protection, lipid metabolism, and gut‐brain axis communication. Therefore, achieving a dynamic balance and diversity in gut microbial composition is essential (Chambers et al., 2018; Liang et al., 2023). It has been discovered that decreasing the pH within the colon can impede the excessive growth of detrimental pH‐sensitive bacteria, including Enterobacteriaceae and Clostridium (Duncan et al., 2009). Therefore, improving cerebral infarction gut health through the intake of ACN from *L. ruthenicum* Murray may develop into another avenue for potentially preventing and treating cerebral infarction.

## 5 CONCLUSION

In this study, we collected feces from patients with cerebral infarction. Also, we collected feces from healthy volunteers. We obtained the differences in the flora composition between the two groups by 16S rRNA gene sequencing analysis, which added new evidence for the changes in the composition of the intestinal flora of patients with cerebral infarction. We hope that the changes in the intestinal flora of patients with cerebral infarction can be given more attention in future studies. Meanwhile, the in vitro fermentation products of ACN significantly increased the diversity of the intestinal flora in patients with cerebral infarction, promoted the proliferation of beneficial bacteria (e.g. Bifidobacterium, Allisonella, and Prevotella), and decreased the growth of potentially harmful bacteria (Dialister, Megamonas, and Clostridium) in the intestines of patients with acute cerebral infarction. Clostridium in the gut and increased levels of SCFAs, showing effects similar to prebiotics. In short, ACN may improve the gut microbiota structure in stroke patients. Further studies on the bioactive potential of ACN from *L. ruthenicum* Murray are warranted.

## AUTHOR CONTRIBUTIONS

**Jun Qiu:** Conceptualization (equal); data curation (equal); investigation (equal); methodology (equal); project administration (equal); resources (equal); validation (equal); writing – original draft (equal). **Bin Ye:** Data curation (equal); methodology (equal); project administration (equal); writing – review and editing (equal). **Lei Feng:** Methodology (equal); project administration (equal); software (equal); writing – review and editing (equal).

## FUNDING INFORMATION

This study was supported by the Jining Science and Technology Bureau (2022YXNS137).

## CONFLICT OF INTEREST STATEMENT

No potential conflict of interest was reported by the authors.

## ETHICS STATEMENT

The study was conducted by the Declaration of Helsinki and approved by the Institutional Review Board (or Ethics Committee) of Jining No. 1 People's Hospital (approval no. 2023083).

## Data Availability

The authors do not have permission to share data.
